# Comparisons of Two Bioelectrical Impedance Devices and Manual versus Sensor-Based Short Physical Performance Batteries for Assessment of Muscle Mass and Physical Performance

**DOI:** 10.3390/s23136026

**Published:** 2023-06-29

**Authors:** Jin-Young Min, Kyoung-Bok Min

**Affiliations:** 1 Veterans Medical Research Institute, Veterans Health Service Medical Center, Seoul 05368, Republic of Korea; minjy@bohun.or.kr; 2 Department of Preventive Medicine, College of Medicine, Seoul National University, Seoul 08826, Republic of Korea; 3 Institute of Health Policy and Management, Medical Research Center, Seoul National University, Seoul 03080, Republic of Korea

**Keywords:** sarcopenia, muscle mass, physical function, bioimpedance analysis, validation

## Abstract

The assessment of muscle mass and physical performance is essential for the diagnosis of sarcopenia. This study examined the validity of bioimpedance analysis (BIA) and a sensor-based short physical performance battery (SPPB) device for analyzing appendicular skeletal muscle mass (ASM) and physical performance. Forty-one older adults were measured for ASM and physical performance with two BIA devices (InBody770 vs. T-SCAN PLUS III) and two SPPB devices (manual mSPPB vs. sensor-based sSPPB). Validity statistics included the intraclass correlation coefficient (ICC) and Bland–Altman plots to examine the agreement of data from the BIA (InBody770 vs. T-SCAN PLUS III) and the SPPBs (mSPPB vs. sSPPB). There was a significant ICC for skeletal muscle mass between the T-SCAN PLUS III and InBody770 devices (ICC = 0.8822; *p* < 0.0001). The mSPPB and sSPPB values showed agreement across all components: 0.8654 for the total scores, 0.8879 for the walking speed, 0.8889 for the chair stand, and 0.6863 for the standing balance. No systemic bias was observed between the two methods for the BIA and SPPB devices. Measurements using the T-SCAN PLUS III and sSPPB seem to be highly correlated with the InBody770 and mSPPB devices in older adults and may be valid for assessing muscle mass and physical performance.

## 1. Introduction

Sarcopenia is characterized by the progressive and generalized loss of skeletal muscle mass and strength [1]. It is a critical health risk factor for falls, fractures, physical disabilities, and mortality [1]. Aging is associated with the deterioration of skeletal muscle and physical dysfunction; therefore, the importance of sarcopenia has been described primarily in the older population. Emerging evidence suggests that the development of sarcopenia is associated with conditions that are not only observed in older individuals but that are also persistent in individuals with several chronic conditions, including cardiovascular disease, diabetes mellitus, and dementia [2]. Sarcopenia has recently been officially recognized as a muscle disease with an ICD-10-MC diagnostic code (M62.84) [1].

Screening and ensuring the adequate diagnosis of individuals at risk of sarcopenia are important in relieving adverse health outcomes and their associated economic burden. Several sarcopenia guidelines are available, such as the Asian Working Group for Sarcopenia (AWGS), European Working Group on Sarcopenia in Older People (EWGSOP2), and Sarcopenia Definitions and Outcomes Consortium [1,3,4], most of which combine elements such as muscle mass, muscle strength, and physical performance. In both research and clinical practice, the diagnosis of sarcopenia requires reliable, valid, repeatable, and cost-effective tools and measurements.

For the measurement of muscle mass, dual-energy X-ray absorptiometry (DXA) is considered an alternative to computed tomography for measuring muscle quantity [5]. However, it is expensive, not portable, and influenced by an individual’s hydration status [1]. Bioelectrical impedance analysis (BIA), a simple and noninvasive modality, does not measure muscle mass directly but estimates it based on the electrical conductivity of the body using a conversion equation and is calibrated with reference to DXA-measured lean mass [6]. With technological advances, the measurement of multiple frequencies in BIA has been developed and allows the prediction of (i) intracellular and extracellular water independently and (ii) the phase angle, which is known to decrease with age and height, increasing with greater FFM in men and women [7]. Multifrequency BIA has been a popular alternative and suitable method to assess muscle mass in the diagnosis of sarcopenia when DXA is unavailable. To assess the risk of sarcopenia, appendicular skeletal muscle mass (ASM, as the sum of the lean mass in the arms and legs) is generally used as the skeletal muscle-mass index. Because the ASM is influenced by body size, the EWGSOP2 and 2019 AWGS recommend muscle-mass assessment to diagnose sarcopenia [1,4].

Physical performance is a multidimensional concept that provides an objective assessment of the function of the whole body and involves central and peripheral nervous functions as well as mobility [8]. The Short Physical Performance Battery (SPPB) is a composite outcomes measure of lower limb physical performance with three timed components to assess balance, mobility, and lower limb strength. The SPPB is a recommended test for physical performance and is an integral part of the diagnostic algorithm for sarcopenia in both EWGSOP2 and AWGS consensus definitions [1]. In the SPPB, a cut-off point of ≤8 has high sensitivity for sarcopenia diagnosis [9]. The measurement of the SPPB is quick, easy to administer, and does not require any specialized equipment. It is measured by research staff, with each component measured manually using a stopwatch, which may be subject to possible measurement errors due to within-subject, inter-trial, and inter-rater effects and may also be liable to day-to-day variation due to patient-level factors [10].

This study aimed to validate the usefulness of a new BIA (T-SCAN PLUS III) and a sensor-based SPPB device for analyzing the body composition and physical performance required for the diagnosis of sarcopenia in older individuals. We evaluated the reliability of T-SCAN PLUS III for measuring the ASM by comparing it with InBody770, and the agreement of the sensor-based SPPB (sSPPB) devices with manual SPPB (mSPPB) tests.

## 2. Materials and Methods

### 2.1. Study Population

Forty-one individuals (men (*n* = 27) and women (*n* = 14)) volunteered to participate in the study. A convenience sample was recruited from the Veterans Health Service Medical Center, a hospital located in Seoul, Republic of Korea.

The inclusion criteria were as follows: (1) age ≥ 65 years; (2) no apparent cognitive impairment affecting compliance with the research protocol; and (3) ability to walk without assistance. The exclusion criteria were as follows: (1) inability to walk independently or with limited mobility; (2) history of acute medical and surgical difficulties; (3) wearing implantable medical devices inside the body; and (4) evidence of infectious diseases or wounds on the palms or soles of the feet.

This study complies with the Declaration of Helsinki. All procedures used in this study were approved by the Institutional Review Board (IRB no. BOHUN 2023-01-066). Informed consent was obtained from all subjects involved in the study.

### 2.2. BIA

Body composition was assessed using the InBody770 (InBodyUSA, Cerritos, CA, USA) and T-SCAN PLUS III (Accuniq, Daejeon, Republic of Korea) devices by applying the principle of multifrequency BIA. In InBody770 and T-SCAN PLUS III, the human body is divided into five cylinders to increase the accuracy of the measurement and hand-to-foot BIA is assessed with eight tactile electrodes. Both machines performed 30 impedance measurements at 6 frequencies (1, 5, 50, 250, 500, and 1000 kHz).

Before the test, the participants removed any metal accessories and belts. They were required to stand upright, and their feet were then centered on the electrodes. The hand electrodes were grasped with the arms wide enough so that there was no contact between the arms and the torso. This position was held for the duration of the test. Once the assessment was completed, the participants were instructed to return their hand electrodes and to exit the device.

The appendicular skeletal mass index was calculated as the sum of the lean mass in the arms and legs measured by each multifrequency BIA device (InBody770 or T-SCAN PLUS III) and divided by the height squared (kg/m^2^).

### 2.3. Measurements of SPPB

The SPPB consists of three tests: a short walk at the usual pace of the individual, standing five times from a seated position in a chair, and a hierarchical assessment of standing balance. Participants completed the mSPPB and sSPPB tests simultaneously to evaluate reliability and avoid the need for repeated assessments.

The mSPPB was performed by trained research personnel using a stopwatch (Casio Model HS-3V-1R), and the time for each individual test performance was manually recorded. Time (in seconds) was recorded with a stopwatch with a resolution of 0.01 s. For the 4 m gait speed, participants were instructed to walk 4 m as fast as possible without slipping, and the time required from the start of standing to the arrival point of 4 m was measured with a stopwatch. For the 5-time chair stand-up test, participants were instructed to stand up from the sitting position and sit down five times as quickly as possible, with their arms folded across the chest. Using a stopwatch, trained staff measured the time taken from the start of the sitting position to the end of the fifth standing step. The standing balance test included a side-by-side stand, a semi-tandem stand, and a tandem stand. The participants were timed until they moved, or 10 s had elapsed, whichever occurred first. Participants were instructed to stand with their feet together side by side for approximately 10 s. They were instructed to use their arms, bend their knees, or move their body to maintain balance, but not move their feet. We stopped the stopwatch and said “stop” after 10 s or when the participants stepped out of position or grabbed their arms. Semi-tandem and tandem stands were conducted in the same manner with side-by-side stability.

**Figure 1:**
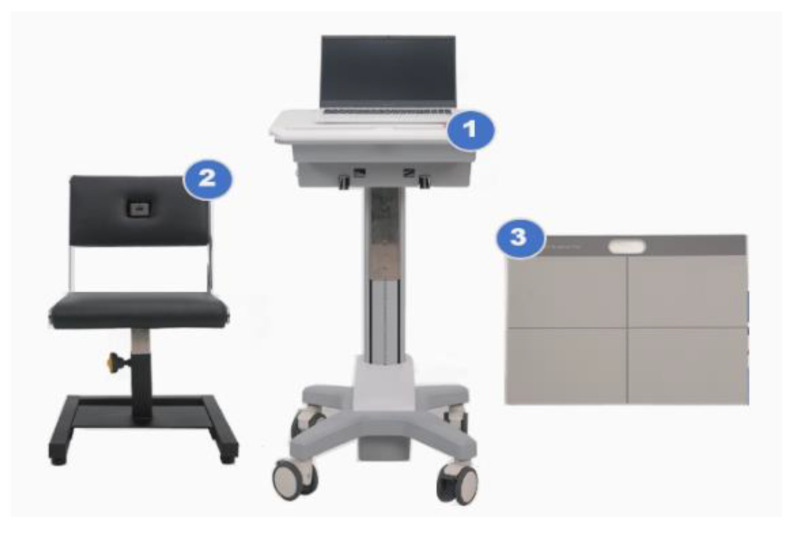
Sensor-based SPPB prototype: (1) laptop and adjustable laptop stand, (2) chair with load cells and LiDAR sensor, and (3) foot mat with load cells.

The sSPPB was measured using the Cyber Medic sSPPB prototype (Gwangju, Republic of Korea) (Figure 1).

The sSPPB was developed to measure three performances (i.e., gait speed, chair stand, and balance tests) to diagnose sarcopenia. The developed device consists of (1) a laptop and adjustable laptop stand, (2) a chair with a load cell and a one-dimensional light-detection ranging (LiDAR) sensor, and (3) a foot mat with digital load cells. The LiDAR sensor is a remote-sensing sensor for distance measurement and object detection. A LiDAR sensor is built into the back of the chair. Load cells are commonly used to measure force or weight, with a maximum measurable load of 150 kg. Load cells are embedded inside the chair seat and the foot mat. For the gait-speed test, a LiDAR sensor is applied to measure the distance between the sensor and the participant. The time spent on the 4 m gait is automatically transmitted to the laptop. For the chair stand-up test, a LiDAR sensor is used to determine the distance between the chair and the person, and load cells measure participant weight. The time spent on the 5-time chair stand-up is automatically transmitted to the laptop. For the standing balance test, load cells built into the foot mat are used to detect the location of the participant’s foot and to measure their weight. The time spent on each position corresponding to a side-by-side stand, a semi-tandem, and a tandem stand is automatically transmitted to the laptop. Each score of the three performances was calculated by the sSPPB algorithm based on previously published cut-off points [11].

### 2.4. Statistical Analysis

To assess the reliability of BIA measurements (InBody770 vs. T-SCAN PLUS III) and the SPPB (mSPPB vs. sSPPB) devices, the sample size was calculated based on the intraclass correlation coefficient (ICC). Zou et al. [12] postulated an ICC of 0.80, with a power (1 − β) of 0.80 and half-width of 95% confidence interval (CI) of ICC < 0.15 [12]. Thirty-five participants were included in the study. We had recruited 41 participants with an anticipated dropout rate of 15%.

For convergent validity, the BIA and SPPB measurement values were compared. We performed Pearson’s correlation and Bland–Altman analyses to evaluate the degree of agreement between the BIA (InBody770 vs. T-SCAN PLUS III) and SPPB (mSPPB vs. sSPPB) devices. The strength of the linear relationship corresponding to the correlation coefficient values was followed in a study by Chen (2003) and defined as very strong (at least 0.8), moderately strong (0.6 up to 0.8), fair (0.3 to 0.5), and poor (less than 0.3) [13]. A Bland–Altman plot with regression analysis is a method of data plotting used to compare differences between mean values and to estimate an agreement limit within a 95% CI [14]. This suggests a possible relationship between the measurements and the true value (i.e., proportional bias), wherein if a proportional bias (*p* < 0.05) exists, the two measurements will not agree equally through the range of the measurements (i.e., the limits of agreement will depend on the actual measurement). We also calculated the ICC of the BIA (InBody770 vs. T-SCAN PLUS III) and the SPPB (mSPPB vs. sSPPB). ICC values below 0.5 indicate a low degree of consent; between 0.5 and 0.75, a medium degree of consent; between 0.75 and 0.9, a high degree of consent; and any value above 0.9 indicates a very high degree of consent. All statistical analyses were performed using Statistical Analysis System software 9.2 (SAS Institute, Cary, NC, USA). Statistical significance was set at *p* ≤ 0.05.

## 3. Results

Table 1 shows the characteristics of the study population. The mean age of the 41 participants was 74.49 years, with two-thirds being male. The average weight, height, and body mass index were 63.49 ± 9.40 kg, 162.02 ± 7.28 cm, and 24.15 ± 2.99 kg/m^2^, respectively.

**Figure 2:**
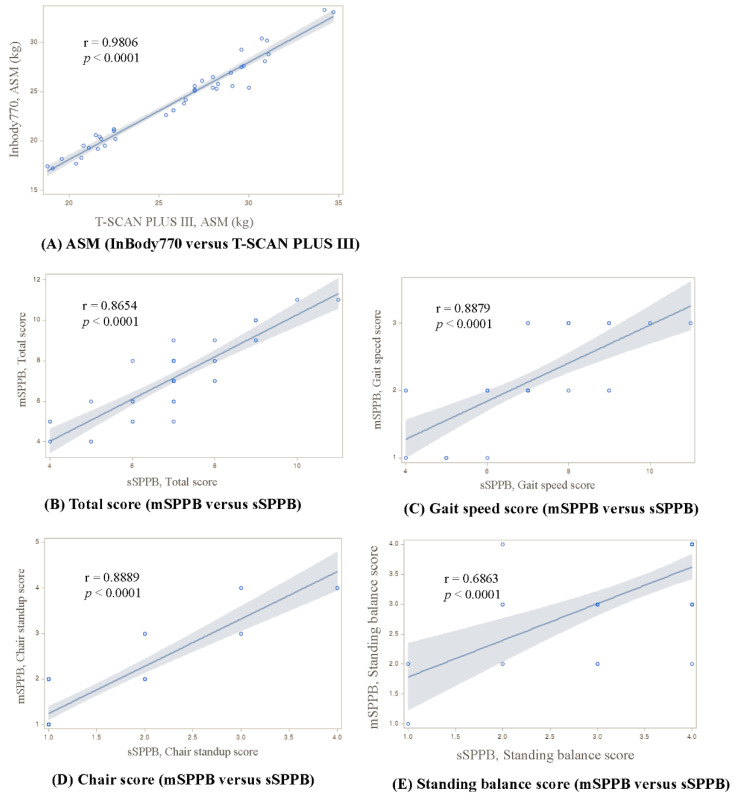
Scatterplots of the correlation of BIA (InBody770 versus T-SCAN PLUS III) and SPPB (mSPPB versus sSPPB) parameters: (**A**) ASM, (**B**) Total score, (**C**) Gait-speed score, (**D**) Chair score, and (**E**) Standing balance score. The blue line represents the linear regression fit, and the gray shading represents the 95% confidence intervals correlation. Within the SPPB parameters, there were identical scores between the mSPPB and the sSPPB, so some points lie on each other or overlap entirely.

The correlations between the BIA and SPPB parameters are shown in Figure 2. The correlation coefficient (*p*-value) of the (A) ASM between InBody770 and T-SCAN PLUS III was very strong at 0.9806 (*p* < 0.0001). Regarding physical performance, SPPB measurements comparing mSPPB and sSPPB were also significantly correlated for all total and component-specific scores: 0.8654 (*p* < 0.0001) for the total SPPB scores (B), 0.8879 (*p* < 0.0001) for the gait-speed scores (C), 0.8889 (*p* < 0.0001) for the chair-stand scores (D), and 0.6863 (*p* < 0.0001) for the balance scores (E).

Table 2 shows the comparison of the two measurement methods in the BIA (InBody770 vs. T-SCAN PLUS III) and SPPB (mSPPB vs. sSPPB). We compared the mean (±SD) values of each parameter and their ICC (95% CI). There was good agreement for the ASM value between InBody770 and T-SCAN PLUS III (ICC = 0.8822; *p* < 0.0001). We further compared the total and component-specific scores of the SPPB, which were of moderate-to-good reliability. ICC values were 0.8181 for the total score, 0.8779 for the gait-speed score, 0.8508 for the chair standup score, and 0.6846 for the standing balance score, and all the values were statistically significant (*p* < 0.0001).

**Figure 3:**
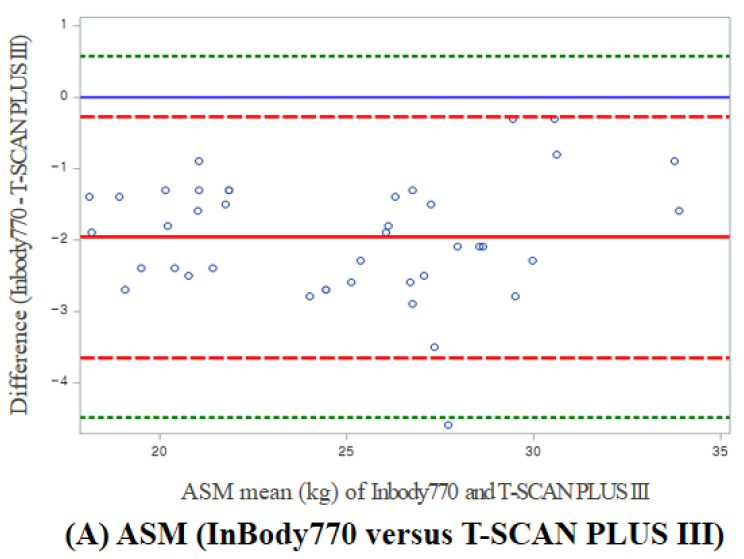

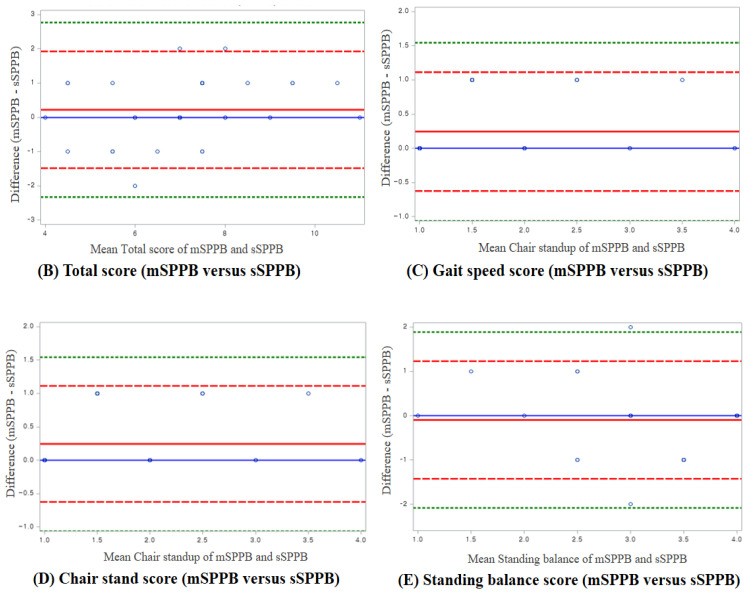
Bland−Altman plots comparing the agreement of BIA (InBody770 versus T-SCAN PLUS III) and SPPB (mSPPB versus sSPPB) parameters: (**A**) ASM, (**B**) Total score, (**C**) Gait-speed score, (**D**) Chair stand score, and (**E**) Standing balance score. The blue line represents the linear regression fit, and the gray shading represents the 95% confidence intervals correlation. Within the SPPB parameters, there were identical scores between the mSPPB and the sSPPB, so some points lie on each other or overlap entirely.

The Bland–Altman plots for each parameter measured using the two different methods for BIA and SPPB are shown in Figure 3. For the ASM measures, the mean difference between the expected percentage (InBody770) and the observed percentage (T-SCAN PLUS III) was negative, but this appeared not to be affected by bias because the data points were evenly distributed along the *x*-axis. For all SPPB parameters, the mean difference was close to 0, and the points were within the 95% agreement limits.

## 4. Discussion

We found that the results from the T-SCAN PLUS III and the sSPPB significantly agreed with that of the InBody770 and mSPPB devices in older adults. This result suggests that the use of T-SCAN PLUS III and sSPPB may be valid methods for evaluating muscle mass and physical performance to diagnose sarcopenia in older adults.

For the diagnosis of sarcopenia, measuring body composition is essential, and BIA, in particular, is a more attractive approach in terms of cost, time, and possible burden to patients than DXA. The definition of sarcopenia is slightly different for each group but is accompanied by a decrease in muscle mass, muscle strength, and physical activity. For the diagnosis of sarcopenia, Baumgartner et al. [15] used DXA and defined sarcopenia as ASM (kg)/height^2^ (m^2^) being less than two standard deviations below the mean of a young reference group. Janssen et al. [16] reported the use of skeletal muscle mass measured using BIA to diagnose sarcopenia based on weight.

Although BIA underestimates fat mass and overestimates fat-free mass relative to DXA, it is a guideline-accepted method for the detection of sarcopenia [17]. As a reference device, InBody770 validated the accuracy of muscle-mass assessment by DXA [18]. This study showed strong correlations for whole-body muscle mass and ASM between DXA analysis and the InBody770 device (r > 0.9; *p* < 0.001). Importantly, in Korea, the application of body composition analysis using multifrequency impedance analysis by region for sarcopenia was approved by the Ministry of Health and Welfare in 2021, indicating the safety and effectiveness of this new health technology to screen for sarcopenia [19].

The multifrequency impedance InBody770 device has been widely applied in several studies related to sarcopenia in clinical and research settings [20,21,22]. The present study compared muscle mass measured using InBody770 (as a reference) and T-SCAN PLUS III. Our data revealed that both devices were highly correlated in measuring ASM (r = 0.9806; *p* < 0.0001). Similar to InBody770, a previous study investigated the reproducibility and validity of T-SCAN PLUS III (note the change in model name from ACCUNIQ BC720 to T-SCAN PLUS III owing to a company name change) compared with DXA [23]. The results showed high reproducibility of repeated measurements of LBM (r = 0.998) and PBF (r = 0.997). The correlation coefficients between ACCUNIQ BC720 and DXA were significantly higher in LBM (r = 0.985) and PBF (r = 0.929). Taken together with the high agreement between T-SCAN PLUS III and InBody770 and the high reproducibility of T-SCAN PLUS III compared with DXA, our data suggest that T-SCAN PLUS III is an interchangeable device for muscle-mass measurements, including ASM, in older adults.

The SPPB is a key measure for clinical outcomes and intervention studies that focus on the physical performance of older adults [8]. The SPPB assesses muscle strength, balance, and mobility through five timed components, all of which are quick, easy to administer, and do not require specialized equipment. However, in the clinical application of the SPPB, it is challenging to reduce the inter-examiner errors of manual SPPB measurements and to ensure the reliability of different SPPB tests, including the 4 m walking, standing five times from a chair, and standing balance tests.

Herein, we compared the performances of two SPPB devices: mSPPB (a typical SPPB measured manually with a stopwatch) and sSPPB (a novel SPPB developed for automatic measurements based on LiDAR and FSR sensors). We observed that the performance time and total scores of each test measured by the mSPPB and the sSPPB were not significantly different and were highly correlated with each corresponding parameter. Our approach is consistent with a prior study conducted by Jung et al. (2019). Those authors developed a multisensor kiosk (eSPPB) and validated its performance by comparing it with a manually measured SPPB [24]. The authors reported significant ICC values for the total score (0.97; *p* < 0.001), gait speed (0.88; *p* < 0.001), the chair stand test (0.99; *p* < 0.001), and standing balance (0.77; *p* < 0.001) between the eSPPB and the mSPPB. They suggest that the newly developed eSPPB kiosk is a valid method to assess physical performance in older adults. Compared with the result of Jung’s study [24], the ICC values between the sSPPB and the mSPPB in our study were 0.87 for the total score, 0.89 for gait speed, 0.89 for the chair-stand test, and 0.69 for the standing balance (0.99; *p* < 0.001)]. The ICC values were comparable for the gait speed but lower for the other test scores. The correlation coefficient assesses the relationship between two variables, while the ICC assesses the agreement between two methods measuring the same variable. When determining the agreement, the values were affected by sample heterogeneity; herein, the variance proportion may originate from participant differences instead of the assessments performed. Our data showed a significant and high degree of consent for parameters between the mSPPB and the sSPPB, but some of the ICC values were relatively low, especially in standing balance. However, the current study also supports the sSPPB as a possible method to estimate the standing balance, gait speed, and standing-on-a-chair test. It can be used to assess physical performance related to the risk of sarcopenia in older adults. Further research is needed to examine the suitability of the sSPPB use in a broader aging population and its feasibility for sarcopenia screening in the clinical setting.

This study has some limitations. First, this was a cross-sectional study, and we were unable to assess the test–retest reliability of T-SCAN PLUS III and the sSPPB. Second, participants were recruited from an outpatient clinic at a single hospital. Our results are limited by the generalizability of the study and may not be applicable to other populations (i.e., frail older adults and young adults). Third, the mSPPB and sSPPB were measured by a single experienced nurse in separate areas. We first measured the mSPPB and then sthe SPPB. This may induce discrepancies in conducting the three timed components to assess the 4 m walking speed, the 5-time stand-up position, and the standing balance between the two methods. Finally, factors (i.e., medication and medical condition) affecting fluid balance and electrical transmission in the BIA assessment were not collected in this study.

## 5. Conclusions

In conclusion, sarcopenia is a severe clinical issue among older adults. Screening individuals at risk of sarcopenia is essential to relieving adverse health outcomes and health-care costs. Our study showed that measurements using the T-SCAN PLUS III and sSPPB were highly correlated with those using the InBody770 and mSPPB devices in older adults and may be valid for assessing muscle mass and physical performance. The current findings should be confirmed to determine whether the use of T-SCAN PLUS III and sSPPB is clinically meaningful for the evaluation of sarcopenia. However, allowing the development of new devices and the comparison of new and existing instruments could be beneficial to actively assessing and coping with sarcopenia in advance.

## Figures and Tables

**Table 1:** Characteristics of the study population (*n* = 41).

Variables	Mean ± SD
Age (years)	74.49 ± 3.57
Male, *n* (%)	27 (65.85%)
Weight (kg)	63.49 ± 9.40
Height (cm)	162.02 ± 7.28
Body mass index (kg/m^2^)	24.15 ± 2.99

**Table 2:** Comparison of BIA and SPPB parameters and ICC (*p*-value).

Bioelectrical Impedance Analysis				
	Inbody770	T-SCAN PLUS III	ICC	(*p*-Value)
ASM (kg)	44.07 ± 7.32	46.12 ± 7.32	0.8822	(<0.0001)
**Physical performance**				
	**mSPPB**	**sSPPB**	**ICC**	**(*p*-value)**
Total score (0–12)	7.24 ± 1.70	7.02 ± 1.47	0.8181	(<0.0001)
Gait speed (0–4)	2.15 ± 0.57	2.07 ± 0.52	0.8779	(<0.0001)
Chair stand-up (0–4)	1.73 ± 0.95	1.49 ± 0.84	0.8508	(<0.0001)
Balance (0–4)	3.37 ± 0.80	3.46 ± 0.80	0.6846	(<0.0001)

Values are presented as mean ± standard deviation. SPPB, Short Physical Performance Battery; ICC, intraclass correlation coefficient; sSPPB, sensor-based Short Physical Performance Battery; mSPPB, manual Short Physical Performance Battery.

## Data Availability

The data that support the findings of this study are available from the corresponding author, K.B.M., upon reasonable request.
