# Correction: Min, J.-Y.; Min, K.-B. Comparisons of Two Bioelectrical Impedance Devices and Manual versus Sensor-Based Short Physical Performance Batteries for Assessment of Muscle Mass and Physical Performance. *Sensors* 2023, *23*, 6026

**DOI:** 10.3390/s24062003

**Published:** 2024-03-21

**Authors:** Jin-Young Min, Kyoung-Bok Min

**Affiliations:** 1 Veterans Medical Research Institute, Veterans Health Service Medical Center, Seoul 05368, Republic of Korea; minjy@bohun.or.kr; 2 Department of Preventive Medicine, College of Medicine, Seoul National University, Seoul 08826, Republic of Korea; 3 Institute of Health Policy and Management, Medical Research Center, Seoul National University, Seoul 03080, Republic of Korea

## Text Correction

There were five errors in the original article [1].

1. In Abstract, instead of “Forty-one older adults were measured for ASM and physical performance with two BIA devices”, it should read: “Forty older adults were measured for ASM and physical performance with two BIA devices”.
2. Section 2.1, Study Population, Paragraph 1, instead of “Forty-one individuals (men (*n* = 27) and women (*n* = 14)) volunteered”, it should read: “Forty individuals (men (*n* = 26) and women (*n* = 14)) volunteered”.
3. Section 2.1, Study Population, Paragraph 3, and Institutional Review Board Statement section, instead of “All procedures used in this study were approved by the Institutional Review Board (IRB no. BOHUN 2023-01-066)”, it should read: “(IRB no. BOHUN 2023-02-014)”.
4. Section 2.4, Statistical Analysis, Paragraph 1, instead of “We had recruited 41 participants with an anticipated dropout rate of 15%”, it should read: “We had recruited 40 participants with an anticipated dropout rate of 11%”.
5. There was a mistake in the published version of Table 1. The number of study population should be “40” instead of “41”. The mean + SD for the males should be “26 (65.00%)” instead of “27 (65.85%)”.

**Table 1:** Characteristics of the study population (*n* = 40).

Variables	Mean ± SD
Age (years)	74.49 ± 3.57
Male, *n* (%)	26 (65.00%)
Weight (kg)	63.49 ± 9.40
Height (cm)	162.02 ± 7.28
Body mass index (kg/m^2^)	24.15 ± 2.99

The authors apologize for any convenience caused and state that the scientific conclusions are unaffected. This correction was approved by the Academic Editor. The original article has been updated.
